# The mayo adhesive probability score predicts postoperative fever and sepsis in retrograde intrarenal surgery

**DOI:** 10.1007/s00240-024-01586-z

**Published:** 2024-05-31

**Authors:** Tsung-Yi Hsieh, Shang-Jen Chang, Jeff Shih-Chieh Chueh, Yuan-Ju Lee

**Affiliations:** Department of Urology, National Taiwan University Hospital, 7 Chung Shan S. Road (Zhongshan S. Road), Zhongzheng Dist, Taipei, 100225 Taiwan (R.O.C.)

**Keywords:** Retrograde intrarenal surgery, Urolithiasis, Mayo adhesive probability score, Postoperative complications, Urinary tract infection, Sepsis

## Abstract

Infectious complications are among the most common and potentially life-threatening morbidities of retrograde intrarenal surgery (RIRS). Few predictive tools on these complications include radiological signs. The Mayo adhesive probability (MAP) score is an image-based scoring system that incorporates two radiological signs: perinephric fat stranding and perinephric fat thickness. Previous studies have suggested an association between these signs and febrile urinary tract infection (UTI) following lithotripsy. This study aimed to evaluate the predictive factors, including the MAP score, for post-RIRS fever and sepsis. A total of 260 patients who underwent 306 RIRS between October 2019 to December 2023 due to renal or upper ureteral stones were included in this retrospective study. Patient demographics, perioperative characteristics, stone factors, radiological signs, and MAP scores were recorded. Multivariate logistic regression analysis was used to evaluate the risk factors associated with postoperative fever and sepsis. Postoperative fever and sepsis occurred in 20.8% and 8.5% of the patients, respectively. On multivariate analysis, female gender, history of recurrent UTI, larger maximal stone diameter, and higher MAP score were independent risk factors for postoperative fever and sepsis. Identifying the risk factors for post-RIRS infectious complications is imperative to providing the proper perioperative management. The MAP score is a promising, easily calculated, image-based scoring system that predicts post-RIRS fever and sepsis.

## Introduction

Urolithiasis is a growing global health concern with an increasing incidence. It affects up to 13% of people during their lifetime, leading to a significant burden on the health care system [1–3]. Contemporary surgical management for renal and upper ureteral stones include extracorporeal shockwave lithotripsy (SWL), percutaneous nephrolithotripsy (PNL), and retrograde intrarenal surgery (RIRS) [4, 5]. RIRS is generally considered safe, minimally invasive, and effective. RIRS removes stones though the natural orifice and has comparable efficacy to PNL except in cases with large renal stones. In addition, RIRS yields a higher stone-free rate than SWL. Hence, there is a notable trend among urologists to prefer RIRS for treating kidney stones compared to other available treatment options [6–8]. However, RIRS has a considerably high complication rate of 9–25%, which include pain, infection, hematuria, ureteral injuries, infection, and other rare complications [5]. In particular, despite the appropriate use of antibiotic prophylaxis and sterile procedures, fever and urinary tract infection (UTI) are the most common complications, and may further progress to sepsis [2, 9–12], which is the leading cause of mortality for nephrolithiasis [13, 14]. Given the potential life-threatening nature of these infectious complications, it is important to identify the predictive factors for postoperative fever and sepsis. Previous studies on the risks of infectious complications following RIRS mostly focused on patient co-morbidities, perioperative clinical presentations, and stone factors [2, 9–12], but few have included radiological signs in their analysis.

The Mayo adhesive probability (MAP) score is a validated image-based scoring system that was developed to predict adherent perinephric fat (APF). It includes two radiological factors, perinephric fat stranding (PFS) type and posterior perinephric fat thickness [15, 16]. PFS, a linear or curvilinear area of soft tissue attenuation in the perirenal fat space, and perirenal edema have been found to be associated with pyelonephritis, bacteremic UTI, acute ureteral obstruction, renal infection, and inflammation [17–23]. Perinephric fat thickness is a reliable measure of the mass of perinephric adipose tissue (PRAT) [24], which has important roles in local and systemic immunoregulation [25]. The aim of the study is to evaluate the predictive value of the MAP score in postoperative fever and postoperative sepsis in patients who underwent RIRS.

## Patients and methods

This study was approved by the institutional review board of our hospital. We retrospectively reviewed all consecutive patients who underwent RIRS from October 2019 to December 2023 due to renal or upper ureteral stones at a tertiary referral center. Patients with anatomical anomalies, pediatric patients (< 18 years old), patients without preoperative CT, and patients who received concurrent contralateral or ipsilateral PNL were excluded. Patient demographics and characteristics, including age, gender, body mass index, and comorbidity, and perioperative clinical parameters, such as blood test, urinalysis, urine culture, duration of antibiotic use, presence of preoperative renal drainage, stone number, maximal stone diameter, procedural laterality, and operative time, were collected.

**Fig. 1:**
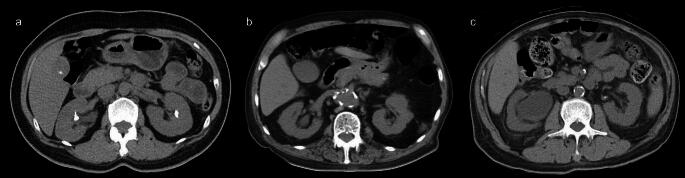
Mayo adhesive probability (MAP) score (**a**) MAP score = 0. The fat around the kidney demonstrates no stranding. The posterior perinephric fat thickness is < 1 cm. (**b**) MAP score = 3. The fat around the kidney has some image-dense stranding present but no thick bars of inflammation. The posterior perinephric fat thickness is 1.1–1.9 cm. (**c**) MAP score = 5. The fat around the kidney shows severe stranding around the kidney with thick image-dense bars of inflammation. The posterior perinephric fat thickness is > 2 cm

All the patients had at least one abdominal CT before the surgery. The key CT was the most recent CT within 3 months prior to RIRS. No stone management was performed during the period between the CT and RIRS. The stone size was determined by measuring the longest axis or the sum of the longest axes in cases of multiple stones on preoperative radiologic investigation [26]. The presence of preoperative hydronephrosis was graded according to the Society for Fetal Urology Hydronephrosis Grading System [27]. Stone clearance was assessed via a postoperative plain abdominal film. Residual stones were defined as fragments greater than 4 mm in size on imaging. Perinephric fat thickness was measured at the level of the renal vein in the axial plane. The lateral perinephric fat thickness was measured from the renal capsule to the sidewall in parallel to the renal vein laterally. The posterior perinephric fat thickness was measured as a direct line posteriorly from the renal capsule to the abdominal wall. PFS was categorized according to the previous literature on stranding. Cases without fat stranding were categorized as none; cases with a few visible thin strands were categorized as mild; cases with many visible thick bands were categorized as severe; and cases between mild and severe were categorized as moderate [28]. The MAP score was calculated from the sum of the posterior perinephric fat thickness score (1 cm = 0 points, 1.1–1.9 cm = 1 point, > 2.0 cm = 2 points) and the PFS stranding type (no stranding = 0 points, mild/moderate = 2 points, severe = 3 points) (Fig. 1) [15].

Recurrent urinary tract infections (rUTIs) were defined as 2 episodes of symptomatic cystitis within the last 6 months or 3 episodes within the previous year. Postoperative febrile UTI was defined as the presence of high fever (> 38 °C) with pyuria, with or without a positive urine culture, and without other infectious signs not associated with UTI within a week of RIRS. Pyuria was defined as the presence of ≥ 10 white blood cells per high power field in a centrifuged urine specimen. Positive urine culture was defined as the presence of ≥ 10^3^ colony-forming units per ml of a single urinary pathogen in the urine sample. Sepsis was defined according to the Third International Consensus Definitions for Sepsis and Septic Shock (Sepsis-3) as life-threatening organ dysfunction caused by a dysregulated host response to infection; organ dysfunction was defined as an acute change in total Sequential Organ Failure Assessment Score ≥ 2 points consequent to the infection. Septic shock was defined as sepsis with persisting hypotension requiring vasopressors to maintain mean arterial pressure ≥ 65 mmHg and having a serum lactate level > 2 mmol/L despite adequate volume resuscitation [29].

In general, preoperative renal drainage may be performed in an obstructed kidney at the discretion of the urologist if the patient presents with hydronephrosis and was at risk of sepsis development or had deteriorating renal function. Surgery was then performed after resolution of the renal infection. Before the operation, patients received a single dose of intravenous prophylactic antibiotics (2nd generation cephalosporin) if no pyuria and a negative urine culture were present. High risk patients, defined as patients with pyuria, positive urine cultures within two weeks of the procedure, or patients with ureteral stents or percutaneous nephrostomy, received an extended course (> 1 day) of preoperative intravenous empirical or sensitive antibiotics according to the most recent urine culture results.

RIRS procedures were performed by multiple experienced endourologist, including experts with > 100 cases, and assisted by urology residents. All RIRS procedures were performed under general anesthesia in the lithotomy position. Before RIRS, ureteroscopy (URS) was performed using a 6/7.5 Fr. semi-rigid ureteroscope (Richard Wolf, Knittlingen, Germany) to assist in dilation and assess ureteral permeability. A 0.035 in. hydrophilic guidewire (Terumo, Tokyo, Japan) was placed under visual guidance. A 10/12 Fr. or 12/14 Fr. ureteral access sheath (AnQing Medical, Shanghai, China or Rocamed, Monaco, Monaco) was introduced through the guidewire to facilitate RIRS.

RIRS was performed using a single-use 7.5 Fr. flexible ureteroscope (Innovex, AnQing Medical, Shanghai, China or Uscope, Pusen Medical, Guangdong, China). A systematic inspection of the pelvicalyceal system was conducted before stone treatment. The stones were fragmented by holmium: yttrium-aluminium-garnet (YAG) lithotripsy using a 270 μm reusable laser fiber (Boston Scientific, Marlborough, MA, USA). A 2.4 Fr. zero-tipped nitinol stone basket (Cook Medical, Bloomington, IN, USA) may be used to facilitate removal of stone fragments. Irrigation pressure during RIRS was maintained below 150 cmH_2_O. At the end of the stone treatment, the collecting system was inspected systematically to confirm adequate lithotripsy. After lithotripsy, a 6 Fr. or 7 Fr. double-J stent (Boston Scientific, Marlborough, MA, USA) was placed endoscopically, which was removed at around postoperative 1 month. Choice of disposable equipment, ureteral access sheath size, laser settings, use of stone basket, and double-J stent size were left to the discretion of the surgeon.

The distributions of demographic and clinical characteristics of the patients were described as median with interquartile range (IQR) for continuous variables and as frequency with percentages (%) for categorical variables. Receiver operating characteristic (ROC) curve analysis was used to define the cut-off value of MAP score for predicting postoperative fever and postoperative sepsis. Logistic regression analysis was used to determine the risk factors associated with postoperative fever and postoperative sepsis. For the multivariate analysis, a stepwise multiple logistic regression model with variable inclusion if *P* < 0.05 and exclusion if *P* > 0.1 was used. A p-value of < 0.05 was considered statistically significant. All data were analyzed using MedCalc Statistical Software version 22.014 (MedCalc Software; http://www.medcalc.org; 2023).

## Results

In all, 364 consecutive patients who received RIRS due to renal or upper ureteral stones were assessed for eligibility. After the exclusion of 104 patients who did not meet the inclusion criteria, 260 patients comprising 111 (42.7%) females and 149 (57.3%) males who underwent a total of 306 RIRS between October 2019 and December 2023 at a single tertiary referral center were included in this study. The demographics and perioperative characteristics of the included patients are listed in Table 1. The median age of the patients was 64.5 (IQR 56–71) years. Comorbid diabetes mellitus was found in 61 (23.5%) patients and comorbid chronic kidney disease was found in 21 (8.1%) patients. Twenty-one (8.1%) patients had a history of rUTI. The median preoperative estimated glomerular filtration rate (eGFR) was 70.2 (IQR 53.8–89.0) ml/min/1.73 m^2^. Preoperative pyuria was found in 158 (60.8%) patients and positive preoperative urine culture was found in 96 (36.9%) patients. Single dose preoperative antibiotic prophylaxis was administered to 85 (32.7%) patients, while 175 (67.3%) patients received an extended course of preoperative antibiotic. Preoperative renal drainage was performed on 41 (13.4%) patients, including 23 cases of ureteral stents and 19 cases of percutaneous nephrostomy. Unilateral RIRS was performed on 213 (81.9%) patients, while bilateral RIRS was performed on 47 (18.1%) patients. The median operative time was 86.5 (IQR 62.5–121) min. The median maximal stone diameter was 12.8 (IQR 8.6–17.5) mm and the median number of stones was 2 (IQR 1–4). Postoperative residual stones were noted in 48 (15.7%) cases (Table 1).

ROC curve analysis was used to evaluate the predictive value of the MAP score on postoperative fever and postoperative sepsis, which showed that the area under the curve (AUC) were 0.798 and 0.799, respectively. At an optimal cut-off value for predicting postoperative fever, MAP score ≥ 3 exhibited a 73.9% sensitivity and 76.8% specificity. The optimal cut-off value for predicting postoperative sepsis was also MAP score ≥ 3, which exhibited an 81.5% sensitivity and 70.6% specificity. MAP scores ≥ 3 were found in 95 (36.5%) patients. Older age, comorbid diabetes mellitus, lower preoperative eGFR, preoperative pyuria, extended preoperative antibiotics course, preoperative hydronephrosis, preoperative renal drainage, longer operative time, larger maximal stone diameter, postoperative fever, postoperative sepsis, and postoperative septic shock were more likely to present in patients with MAP scores ≥ 3 compared to patients with MAP scores < 3 (Table 1).

**Table 1:** Perioperative characteristics of patients according to MAP score < 3 and MAP score ≥ 3

Characteristics	All	MAP score < 3	MAP score ≥ 3	*P*-value
Patients, *n* (%)	260	165 (63.5)	95 (36.5)	
Age, year, median (IQR)	64.5 (56–71)	63 (52–70)	67 (60–73)	< 0.001*
Female gender, *n* (%)	111 (42.7)	71 (43.0)	40 (42.1)	0.884
Body mass index, kg/m^2^, median (IQR)	25.3 (23.0–28.4)	25.1 (22.3–28.1)	25.5 (23.3–29.1)	0.179
Diabetes mellitus, *n* (%)	61 (23.5)	32 (19.4)	29 (30.5)	0.042*
Hypertension, *n* (%)	115 (44.2)	70 (42.4)	45 (47.4)	0.440
Chronic kidney disease, *n* (%)	21 (8.1)	11 (6.7)	10 (10.5)	0.272
Recurrent urinary tract infections, *n* (%)	21 (8.1)	10 (6.1)	11 (11.6)	0.117
Preoperative eGFR, mL/min/1.73 m^2^, median (IQR)	70.2 (53.8–89.0)	76.0 (59.0–91.1)	63.1 (47.2–85.9)	< 0.001*
Preoperative neutrophils count, 10^3^ cells/µl, median (IQR)	4.26 (3.29–5.22)	4.16 (3.27–4.98)	4.46 (3.40–5.95)	0.103
Preoperative pyuria, *n* (%)	158 (60.8)	88 (53.3)	70 (73.7)	0.001*
Preoperative positive urine culture, *n* (%)	96 (36.9)	55 (33.3)	41 (43.2)	0.115
Preoperative antibiotic prophylaxis				0.001*
Single dose, *n* (%)	85 (32.7)	66 (40.0)	19 (20.0)	
Extended course, *n* (%)	175 (67.3)	99 (60.0)	76 (80.0)	
Preoperative hydronephrosis				< 0.001*
No hydronephrosis, *n* (%)	116 (37.9)	91 (45.0)	25 (24.0)	
Grade 1–2, *n* (%)	69 (22.5)	44 (21.8)	25 (24.0)	
Grade 3–4, *n* (%)	121 (39.5)	67 (33.2)	54 (51.9)	
Preoperative renal drainage	41 (13.4)	17 (10.3)	24 (25.3)	< 0.001*
Ureteral stent, *n* (%)	23 (7.5)	11 (6.7)	12 (12.6)	
Percutaneous nephrostomy, n (%)	19 (6.2)	6 (3.6)	13 (13.7)	
Procedural laterality				0.542
Unilateral, *n* (%)	213 (81.9)	137 (83.0)	76 (80.0)	
Bilateral, *n* (%)	47 (18.1)	28 (17.0)	19 (20.0)	
Operative time, minutes, median (IQR)	86.5 (62.5–121)	80 (60–115.5)	102 (71.3–125.5)	0.015*
Maximal stone diameter, mm, median (IQR)	12.8 (8.6–17.5)	11.8 (7.90–17.0)	13.7 (11.12–18.1)	0.008*
Number of stones, median (IQR)	2 (1–4)	2 (1–3)	3 (1–4)	0.054
Stone radiodensity, Hounsfield unit, median (IQR)	703 (516–939)	693 (511–930)	719 (531–975)	0.519
Postoperative residual stones, *n* (%)	48 (15.7)	28 (13.9)	20 (19.2)	0.222
Postoperative fever, *n* (%)	54 (20.8)	14 (8.5)	40 (42.1)	< 0.001*
Postoperative sepsis, *n* (%)	22 (8.5)	4 (2.4)	18 (18.9)	< 0.001*
Postoperative septic shock, *n* (%)	8 (3.1)	2 (1.2)	6 (6.3)	0.022*
Perinephric fat stranding				< 0.001*
No stranding, *n* (%)	176 (57.5)	176 (87.1)	0 (0)	
Mild stranding, *n* (%)	66 (21.6)	20 (9.9)	46 (44.2)	
Moderate stranding, *n* (%)	32 (10.5)	6 (3.0)	26 (25.0)	
Severe stranding, *n* (%)	32 (10.5)	0 (0)	32 (30.8)	
Posterior perinephric fat thickness, mm, median (IQR)	12.0 (7.8–18.2)	9.4 (6.3–14.8)	17.6 (13.3–22.8)	< 0.001*
Lateral perinephric fat thickness, mm, median (IQR)	18.4 (12.6–24.0)	16.6 (11.3–22.2)	21.3 (16.3–26.1)	< 0.001*

IQR, interquartile range; eGFR, estimated glomerular filtration rate; MAP score, Mayo adhesive probability score.

*Statistically significant.

### Postoperative fever

Postoperative fever developed in 54 (20.8%) patients (Table 1). Univariate analysis revealed that postoperative fever was significantly associated with the female gender, history of rUTIs, preoperative pyuria, extended preoperative antibiotics course, preoperative SFU grade 3–4 hydronephrosis, longer operative time, larger maximal stone diameter, postoperative residual stones, and higher MAP score. On multivariate logistic regression analysis, independent risk factors for postoperative fever were the female gender (Odds ratio [OR] 2.21, *P* = 0.027), history of rUTIs (OR 3.44, *P* = 0.020), larger maximal stone diameter (OR 1.07, *P* = 0.001), and higher MAP score (OR 2.24, *P* < 0.001) (Table 2).

**Table 2:** Risk factors associated with postoperative fever

	Univariate				Multivariate		
	OR	95% CI	*P*-value		OR	95% CI	*P*-value
Age	1.02	[0.99, 1.04]	0.190				
Female gender	1.91	[1.10, 3.32]	0.022		2.21	[1.09, 4.47]	0.027
Body mass index	1.00	[0.94, 1.06]	0.958				
Diabetes mellitus	1.30	[0.70, 2.41]	0.414				
Hypertension	0.93	[0.53, 1.61]	0.790				
Chronic kidney disease	1.50	[0.60, 3.75]	0.391				
Recurrent urinary tract infections	4.86	[2.21, 10.71]	< 0.001		3.44	[1.22, 9.72]	0.020
Preoperative eGFR	1.00	[0.99, 1.01]	0.460				
Preoperative neutrophils count	1.02	[0.95, 1.10]	0.599				
Preoperative pyuria	2.45	[1.30, 4.60]	0.006				
Preoperative positive urine culture	1.49	[0.85, 2.61]	0.159				
Preoperative extended antibiotics course	3.03	[1.51, 6.09]	0.002				
Preoperative hydronephrosis							
No hydronephrosis	1 (Reference)						
Grade 1–2	1.51	[0.71, 3.24]	0.287				
Grade 3–4	1.96	[1.03, 3.73]	0.041				
Preoperative renal drainage	1.23	[0.57, 2.67]	0.597				
Procedural bilaterality	1.20	[0.67, 2.15]	0.538				
Operative time	1.01	[1.00, 1.01]	0.024				
Maximal stone diameter	1.06	[1.03, 1.10]	< 0.001		1.07	[1.03, 1.11]	0.001
Number of stones	1.07	[0.93, 1.22]	0.335				
Stone radiodensity	1.00	[1.00, 1.00]	0.738				
Postoperative residual stones	2.13	[1.08, 4.19]	0.028				
Perinephric fat stranding							
No stranding	1 (Reference)						
Mild stranding	5.40	[2.54, 11.46]	< 0.001				
Moderate stranding	6.94	[2.82, 17.08]	< 0.001				
Severe stranding	14.88	[6.13, 36.10]	< 0.001				
Posterior perinephric fat thickness	1.06	[1.02, 1.10]	< 0.001				
Lateral perinephric fat thickness	1.02	[0.99, 1.05]	0.190				
MAP score	2.20	[1.76, 2.74]	< 0.001		2.24	[1.76, 2.84]	< 0.001

OR, odds ratio; CI, confidence interval; eGFR, estimated glomerular filtration rate; MAP score, Mayo adhesive probability score

### Postoperative sepsis

Postoperative sepsis developed in 22 patients (8.5%). Among them, septic shock developed in 8 (3.1%) patients (Table 1). Univariate analysis revealed that postoperative sepsis was associated with the female gender, history of rUTIs, extended preoperative antibiotics course, longer operative time, larger maximal stone diameter, postoperative residual stones, and higher MAP score. On multivariate logistic regression analysis, independent risk factors for postoperative sepsis were the female gender (OR 7.11, *P* < 0.001), larger maximal stone diameter (OR 1.07, *P* = 0.009), and higher MAP score (OR 2.49, *P* < 0.001) (Table 3).

**Table 3:** Risk factors associated with postoperative sepsis

	Univariate				Multivariate		
	OR	95% CI	*P*-value		OR	95% CI	*P*-value
Age	1.02	[0.98, 1.05]	0.298				
Female gender	4.32	[1.77, 10.57]	0.001		7.11	[2.60, 19.42]	< 0.001
Body mass index	0.95	[0.86, 1.05]	0.311				
Diabetes mellitus	1.39	[0.58, 3.31]	0.463				
Hypertension	0.79	[0.35, 1.76]	0.561				
Chronic kidney disease	1.46	[0.41, 5.24]	0.561				
Recurrent urinary tract infections	6.48	[2.58, 16.25]	< 0.001				
Preoperative eGFR	0.99	[0.98, 1.01]	0.204				
Preoperative neutrophils count	1.00	[0.88, 1.12]	0.958				
Preoperative pyuria	1.86	[0.76, 4.54]	0.174				
Preoperative positive urine culture	1.77	[0.80, 3.92]	0.159				
Preoperative extended antibiotics course	4.47	[1.31, 15.21]	0.017				
Preoperative hydronephrosis							
No hydronephrosis	1 (Reference)						
Grade 1–2	1.52	[0.53, 4.41]	0.436				
Grade 3–4	1.49	[1.03, 3.78]	0.405				
Preoperative renal drainage	1.14	[0.37, 3.47]	0.821				
Procedural bilaterality	1.37	[0.60, 3.11]	0.458				
Operative time	1.01	[1.00, 1.02]	0.031				
Maximal stone diameter	1.06	[1.02, 1.11]	0.005		1.07	[1.02, 1.12]	0.009
Number of stones	1.07	[0.89, 1.30]	0.459				
Stone radiodensity	1.00	[1.00, 1.00]	0.077				
Postoperative residual stones	4.50	[1.94, 10.44]	< 0.001				
Perinephric fat stranding							
No stranding	1 (Reference)						
Mild stranding	3.42	[1.01, 11.62]	0.049				
Moderate stranding	7.89	[2.25, 27.73]	0.001				
Severe stranding	15.55	[4.87, 49.67]	< 0.001				
Posterior perinephric fat thickness	1.05	[1.00, 1.10]	0.037				
Lateral perinephric fat thickness	1.02	[0.98, 1.06]	0.415				
MAP score	2.18	[1.59, 3.00]	< 0.001		2.49	[1.71, 3.61]	< 0.001

OR, odds ratio; CI, confidence interval; eGFR, estimated glomerular filtration rate; MAP score, Mayo adhesive probability score

## Discussion

In recent years, RIRS has seen increasing preference in the treatment of upper ureteral and renal stones due to its minimally invasive nature, effectiveness, and safety. It has become the treatment of choice for renal stones up to 20 mm and is often considered as an option for treating even bigger stones [4–8, 30, 31]. However, postoperative fever and the more clinically serious sepsis, though rare, may still occur. The recent EAU guidelines report a post-URS sepsis rate up to 5% [5]. However, the incidence of infectious complications of RIRS may be even higher due to differences in surgery-related risk factors, such as higher irrigation pressure during RIRS compared to semirigid URS [2]. Corrales M et al. reported a post-RIRS sepsis ranging from 0.5 to 11.1% [2]. The wide range of incidence may reflect the heterogeneity of patient-related factors and lack of consensus on surgical protocols among studies. Our postoperative sepsis rate of 8.5% falls in line with the findings of Corrales M et al. and other studies [2, 5, 9–12]. Still, the relatively high incidence of these infectious complications, including the high 20.8% postoperative fever rate, may be partly explained by the high proportion of patients with upper ureteral stones or renal stones, which have been associated with postoperative infectious complications [2].

Our study found that the MAP score was an independent risk factor for postoperative infectious complications. Patients with MAP scores ≥ 3 are significantly more likely to develop postoperative fever, postoperative sepsis, and postoperative septic shock. The MAP score is an easily calculated, well-validated scoring system that incorporates PFS and perinephric fat thickness to predict the presence of APF [15]. Though initially developed for partial nephrectomy, the MAP score may be adapted to predict postoperative complication in the management of nephrolithiasis. Chen WA et al. found that a high MAP score was associated with postoperative fever in patients undergoing mini-PNL [32]. The association between the MAP score and infectious complications of RIRS is consistent with previous studies evaluating the relationship between secondary signs on preoperative CT and febrile UTI after URS [21]. Hydronephrosis, enlarged ureter, PFS, and perirenal fat thickening are secondary signs on CT that suggest ureteral obstruction. PFS represents fluid collection within the bridging septa of the perinephric fat due to an increase in intrapelvic pressure. The increased intrapelvic pressure causes an increase in lymphatic pressure, which in turn leads to fluid diffusion into the renal interstitium and subsequent rupture of the renal calyces and perinephric edema [21–23]. Increased renal pressure has also been shown to be a risk factor for post-RIRS sepsis [2]. Perinephric fat thickness is a reliable measure of PRAT mass and APF [24]. Factors including fibrosis, immune response, and inflammation may contribute to the complex underlying pathogenesis of APF [15, 22, 23]. In particular, the immunoregulatory adipokines and cytokines produced by PRAT, such as interleukin-6, IL-1β, and TNF-α, may have local and systemic pro-inflammatory effects [24, 25]. Thus, excessive PRAT, when triggered, may lead to an increased inflammatory response, which predisposes patients to postoperative fever or sepsis [25].

Consistent with previous studies that evaluated the risk factors for infectious complications, we found that the female gender, history of rUTIs, and larger stone size were independent risk factors for post-RIRS infectious complications [2, 9–12]. The higher rate of infectious complications in women may be attributed to the shorter urethral length of women and increased risk of colonization by perineal and rectal bacteria [2]. A larger stone size or greater stone burden may be explained by the larger stones having a higher bacterial load and greater endotoxin release during stone fragmentation [2, 9, 11]. Of particular note was that rUTI, although an essential comorbidity in its impact on infectious complications, was an independent risk factor for postoperative fever, but not for sepsis. This may be because patients with rUTI tended to receive a longer duration of preoperative antibiotics as well as have a higher MAP score. Thus, its impact may be overshadowed by the other factors.

Among the 95 patients with MAP score ≥ 3, only 11 (11.6%) patients presented with acute pyelonephritis, defined as febrile UTI with associated signs or symptoms, such as flank pain, nausea, or vomiting, upon admission. Of those 11 patients, seven patients received preoperative renal drainage and antibiotics before receiving RIRS. The other four patients who did not receive preoperative renal drainage had either no hydronephrosis in one case or mild hydronephrosis (SFU grade 1–2) in the other three cases. They were treated conservatively with antibiotics before receiving RIRS. Most of the cases with MAP score ≥ 3 and hydronephrosis did not initially present with fever upon admission, and this likely accounts for the relatively few cases of preoperative renal drainage.

The duration of preoperative antibiotic prophylaxis (single dose vs. extended course) was not a risk factor for postoperative febrile UTI or sepsis in the present study. Our preoperative antibiotic therapy was based on the American Urological Association (AUA) and the European Association of Urology (EAU) guidelines and tailored to the individualized potential risks of patients. The AUA guidelines recommend antibiotic prophylaxis administration based on prior urine culture results or the local antibiogram administration prior to stone intervention [33]; the EAU guidelines recommend perioperative antibiotic prophylaxis for all patients undergoing endourological treatments [5]. However, both recommendations are based on limited evidence and a consensus for antibiotic prophylaxis strategy for URS/RIRS has not been reached [5, 33]. Deng T et al. found in a meta-analysis of results from clinical trials on the use of antibiotic prophylaxis in ureteroscopic lithotripsy that preoperative antibiotic prophylaxis did not lower the risk of postoperative febrile UTI [34]. Méndez-Guerrero DM et al. found that in patients with negative urine culture who underwent flexible URS, there were no differences in the incidence of upper UTI or urosepsis between those with extended antibiotic prophylaxis and those with standard antibiotic prophylaxis [35]. Further, in a randomized controlled trial comparing antibiotic prophylaxis regimen in patients with sterile urine receiving RIRS, Zhao Z et al. reported no difference in the incidences of systemic inflammatory response syndrome (SIRS) between patients receiving no antibiotic prophylaxis and those receiving intravenous ciprofloxacin antibiotic prophylaxis regimens. However, the subgroup analysis demonstrated a greater risk of SIRS in patients who did not receive antibiotic prophylaxis when the stone size was > 200 mm^2^ [36], indicating that in select patients, antibiotic prophylaxis may be necessary. Indeed, there is still a lack of studies on perioperative antibiotic regimen, such as the dose and duration, in patients receiving RIRS. More studies are needed to establish the optimal strategy that takes into consideration individual patient characteristics to prevent infectious complications while maintaining antibiotic stewardship.

There are several limitations in the study. First, our results were based on retrospective patient data from a single medical center. Large-scale prospective studies are necessary to validate our findings. Secondly, while the variables evaluated in our analysis were determined by including possible risk factors reported in previous studies, they are limited by the availability of data from retrospective chart reviews. The statistical analysis might have bias inherent to this type of study. For example, while many of our patients presented with preoperative hydronephrosis and perinephric fat stranding, relatively few patients received preoperative renal drainage. However, interpretation of this discrepancy is difficult because the indications for preoperative renal drainage or conservative antibiotics treatment were not well recorded for each patient. Furthermore, indications for the duration of preoperative antibiotics use were also not well documented, limiting our ability to analyze these data more thoroughly. Thirdly, for our stone size metric, we measured the longest axes of stones or the sum of the longest axes in cases of multiple stones based on the Resorlu-Unsal Stone Score for predicting stone free rates [26]. However, there is a great heterogeneity in the definition of stone size or stone burden in the current literature on renal stones. Further standardization of metrics may be needed to reliably compare our findings with other studies and predictive tools. Fourth, most patients did not have a postoperative CT and thus analysis of the changes in radiologic findings before and after RIRS could not be performed.

## Conclusions

The MAP score was a significant predictive factor for postoperative fever and sepsis in patients receiving RIRS. Patients with an MAP score ≥ 3 were significantly more likely to develop infectious complications after RIRS. By incorporating the easily-calculated, image-based MAP score, endourologists can better predict these complications and provide the appropriate perioperative management.

## Data Availability

No datasets were generated or analysed during the current study.
